# Digital Technology for Caregivers of People With Psychosis: Systematic Review

**DOI:** 10.2196/mental.9857

**Published:** 2018-09-05

**Authors:** Juliana Onwumere, Filipa Amaral, Lucia R Valmaggia

**Affiliations:** ^1^ Institute of Psychology, Psychiatry and Neuroscience Department of Psychology King's College, London London United Kingdom; ^2^ Bethlem Royal Hospital South London and Maudsley NHS Foundation Trust London United Kingdom

**Keywords:** carers, digital interventions, families, psychosis, technology

## Abstract

**Background:**

Psychotic disorders are severe mental health conditions that adversely affect the quality of life and life expectancy. Schizophrenia, the most common and severe form of psychosis affects 21 million people globally. Informal caregivers (families) are known to play an important role in facilitating patient recovery outcomes, although their own health and well-being could be adversely affected by the illness. The application of novel digital interventions in mental health care for patient groups is rapidly expanding; interestingly, however, far less is known about their role with family caregivers.

**Objective:**

This study aimed to systematically identify the application of digital interventions that focus on informal caregivers of people with psychosis and describe their outcomes.

**Methods:**

We completed a search for relevant papers in four electronic databases (EMBASE, MEDLINE, PsycINFO, and Web of Science). The search also included the Cochrane database and manual search of reference lists of relevant papers. The search was undertaken in accordance with Preferred Reporting Items for Systematic Reviews and Meta-Analyses reporting guidelines.

**Results:**

The search identified 9 studies derived from 8 unique datasets. Most studies were assessments of feasibility and were undertaken in the United States. Interventions were predominately Web-based, with a focus on improving the caregivers’ knowledge and understanding about psychosis.

**Conclusions:**

This study offers preliminary support for the feasibility and acceptability of digital interventions for psychosis in informal caregiver populations. However, the findings underpin a clear need for greater development in the range of caregiver-focused digital approaches on offer and robust evaluation of their outcomes. The use of digital approaches with caregiver populations seemingly lags someway behind the significant developments observed in patient groups.

## Introduction

### Informal Care Provision

Despite optimal pharmacological interventions, as many as one-third of patients with psychosis might continue to experience persistent psychotic symptoms, as well as comorbid conditions, including depression and anxiety [1,2], implying that across the globe, as care provision for adults living with psychosis conditions continues to devolve from hospitals to community settings, informal (unpaid) caregivers provide the bulk of care and play a central role in affecting treatment outcomes. Informal caregivers are primarily close relatives of patients, such as their parents, siblings, partners, and offspring, and many live with and maintain close regular contact with their relatives, particularly during the early illness course [3,4]. Patients who are supported by informal caregiving relationships tend to have improved functioning and recovery outcomes, including improved life expectancy [5], fewer relapses and need for hospital admissions [6], and higher levels of engagement with and improvements from prescribed treatments [7,8]. The economic value of unpaid caregiver support is estimated at several billion pounds each year [9]; these figures are remarkable in the light of robust findings indicating that high levels of burden are reported by caregivers and at least one-third admit to being at “breaking point” in their role [10,11]. Approximately 40% of caregivers report clinical levels of depression and anxiety [4,12,13]; feelings of loss, grief, and despair are also commonplace [14]. The physical health of caregivers can also be compromised, including experiencing elevated rates of sleep disturbance [15-17].

Interventions to improve caregivers’ understanding of psychosis, facilitate adaptive coping strategies, and provide support and stress management skills have proven efficacy [18,19] and are included in treatment guidelines in many regions, including Canada [20], Australia [21], the United States [22], and the United Kingdom [23]. However, there remains an ongoing issue of how to best increase the provision and access to evidence-based interventions for caregivers. Psychosis caregivers are a neglected group that has independent care, information, and support needs that mental health providers can typically struggle to respond. Reportedly, evidence-based family interventions, like other psychological therapies, are not widely available and rates of implementation in mental health trusts and services can range between 0% and 53% [24,25]. Several barriers to implementation and widening access have been posited, including issues related to family engagement, time demands, and insufficient staff and resources [26]. Hence, a growing need exists to explore options that help to address these obstacles and increase caregiver access to support interventions. Furthermore, the importance of seeking to identify effective and acceptable approaches to responding to the needs of psychosis caregivers is widely acknowledged, given the integral role they play in optimizing patient outcomes. Moreover, as caregivers with poor health status are more likely to relinquish their caregiving role and consequently affect patient outcomes and care costs, the need to focus on caregivers and optimal care provision is axiomatic.

### Digital Interventions in Health Care

An ever-increasing proportion of the world’s adult population is online [27]. In the United Kingdom, for example, 78% of the adult population (approximately 39.3 million) is online each day, with a similar proportion accessing the internet using mobile devices, including mobile phone or portable computers [28]. Searching for information, including those related to health issues, constitute some of the most popular Web-based activities [28,29]. The last decade has witnessed considerable growth and innovation in the application of digital technologies (electronic health), including virtual and augmented reality, to support the assessment, understanding, and treatment of a wide range of health conditions [30-33], including mental health, such as psychosis [34]. These have included, for example, developments with mobile apps (eg, mobile phones) to assess mood functioning and symptoms [35], Web-based psychological therapies [36], interactive short message service text messages [37], computerized interventions [38], and wearable technologies that offer real-time feedback on well-being and functioning such as activity and sleep quality [39,40]. Furthermore, digital interventions could be a useful way to offer time and cost-effective approaches to reach and engage with larger populations, including those who might be less willing or able to access standard services because of geography and travel burden, or where flexible modes of access and privacy are prioritized.

### Study Aims

This study aimed to review the application of digital interventions and their outcomes with families (informal caregivers) of people with psychosis. It specifically aimed to characterize the type of interventions used with caregiver populations and their key components. This study included a broad definition of “digital interventions” to capture any approach designed to affect an individual’s understanding, functioning, behavior, and well-being.

## Methods

### Design

This was a systematic review of the literature with a qualitative synthesis of the findings.

### Selection Procedure

In accordance with the Preferred Reporting Items for Systematic Reviews and Meta-Analyses statement [41] we searched four electronic databases (EMBASE, MEDLINE, PsycINFO, and Web of Science) from inception to June 30, 2017. The search included the Cochrane database and manual search of the reference list of relevant papers.

Studies eligible for review were as follows: (1) those reporting the application and outcomes of a digital (ie, internet-, mobile-, virtual or augmented reality-, telephone-, or app-based) intervention; (2) those having target population including informal caregivers (ie, unpaid relatives or friends) of an individual with psychotic disorder; (3) those reporting caregiver-focused outcomes; (4) those published in English; (5) those reported in a peer-reviewed journal. Ineligible studies were those reporting data from a single person case studies and reviews, having samples with 5 or less participants, and including nonpsychotic disorder illness conditions. However, studies using mixed diagnostic groups were included if psychotic disorders constituted at least 50% of the sample. Furthermore, studies using patient and caregiver samples were eligible, although we focused on caregiver outcomes.

### Search Criteria

To increase the search capabilities and accurate selection of papers, a comprehensive list of keywords and Medical Subject Headings, were used along with relevant search truncations and wildcards to capture variations in language and database indexing.

**Figure 1:**
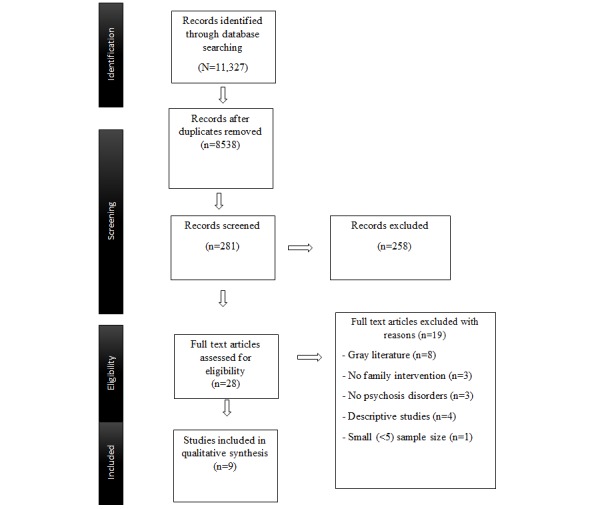
Preferred Reporting Items for Systematic Reviews and Meta-Analyses (PRISMA) flow diagram.

Search terms related first to the digital technologies comprising “virtual reality” OR teleme* OR telemedicine OR telepsychiatry OR telehealth OR eHealth OR mHealth OR “mobile phone” OR “mobile health” OR “mobile technolog*” OR “mobile application” OR smartphone* OR internet OR online OR “online system*” OR “social media” OR “Web-based intervention*” OR “augmented reality” OR e-learning OR computer* OR “computer assisted therapy” OR apps OR “mobile application.” The second group of terms focused on caregivers comprising family OR families OR sibling*OR relative* OR “first-degree relative” OR partner*OR “domestic partner*” OR parent* OR caregiver* OR carer*. The third term related to the psychosis spectrum and included psychos* OR psychotic OR schizophren* OR “at risk mental state” OR “ultra high risk” OR paranoi* OR delusion* OR hallucination*. The Boolean operator AND was used to combine the three primary search term categories.

Initially, the titles and abstracts of identified papers were screened by the second author (FA) against the eligibility criteria to remove duplicates. Subsequently, full-text papers for the remaining papers were obtained where the title and abstract were jointly reviewed by all three authors. Any areas of disagreement between the reviewers about a decision to include or exclude were resolved through discussion. Figure 1 presents the study selection process.

### Assessment of Methodological Quality

The methodological quality of eligible studies was reviewed using the Quality Assessment Tool for Quantitative Studies (QATO) assessment tool [42]. The tool, which has good content and construct validity [43,44], was designed to evaluate the quality of quantitative studies in 6 key domains comprising study design, data collection methods, blinding, selection bias, confounding variables, and withdrawals and dropouts.

Each study was assigned a global rating of methodological quality denoting the overall strength of the ratings across the individual domains. Studies with no weak domain ratings were classified as “strong”; those with at least 1 weak rating were classified as “moderate”; and those with ≥2 weak ratings as “weak.” All papers were rated by author FA, and 50% of papers were rated by at least two authors (FA, LRV, and JO), with any rating discrepancy resolved through discussion.

## Results

### Information Extraction

Our database and manual search methods yielded 11,327 papers, which were subsequently reduced to 8538 studies, following the exclusion of duplicates.

Next, we retrieved 28 full-text papers, which were read in full and assessed against the inclusion or exclusion criteria. Overall, we excluded 19 papers subsequently, resulting in a total of 9 studies, derived from 8 unique datasets, which met full criteria for the inclusion in this review and were assessed against the inclusion criteria by all three authors (FA, JO, and LRV). One paper [45] presents follow-up data from their original study [46]. Figure 1 presents the flowchart detailing the extraction.

### Qualitative Synthesis

In addition to author details, we extracted relevant characteristics from the selected studies on study origin, rationale, design, sample, and details of the intervention and outcomes (Tables 1 and 2).

#### Study Characteristics

##### Study Origin

Regarding the study origin, 4 of the 9 studies were undertaken solely in the United States [45,46,48,52]. Except for one study, the remaining studies were conducted in Hong Kong [47], Ireland [49], Turkey [50], and the United Kingdom [53]. In one study [51], 26 of 30 participants were recruited from the United States and the remaining sample had been recruited from Peru, Australia, and Canada.

##### Participant Characteristics

Across the studies, the total number of caregiver participants was 305. The individual number of participants reported in each study ranged from 16 to 91 [47,52]. Participants were predominately females in 8 of 9 nine studies that provided gender data [45-51,53]. While more than half the studies employed caregiver participants only, 4 papers reported data from caregiver participants and individuals they provided informal care for [45,46,48,52].

##### Study Rationale

There was heterogeneity in the reported study objectives, with two-thirds reporting to be an investigation of the feasibility, acceptability, and usability of the intervention [45-48,52,53]. Two studies specifically sought to test the effectiveness of their intervention [49,50]. In the final paper [51], an online self-help group sought to examine the functionality of the user communications.

**Table 1:** Summary of reviewed studies.

Reference	Origin	Sample	N	Caregiver (%)/gender	QATO^a^ rating	Digital intervention type
Chan et al [47]	Hong Kong	First-episode psychosis caregivers	81	75/F^b^	Weak	Website
Glynn et al [48]	United States	Patients with schizophrenia or schizoaffective disorder living in the community and their relatives	42	83/F	Moderate	Website
Haley et al [49]	Ireland	Relatives of people with a schizophrenia spectrum disorder	56	Not reported	Weak	Telepsychiatry videoconferencing
Ozkan et al [50]	Turkey	Primary caregivers of hospitalized patients with schizophrenia	62	53/F	Moderate	Telepsychiatriy telephone
Perron [51]	United States, United Kingdom, Peru, Australia	Relatives of people with schizophrenia and related mental health problems	33	79/F	Weak	Internet: email and bulletin board
Rotondi et al [46]	United States	Persons with schizophrenia spectrum (n=30) and their informal caregivers	21	68/F	Weak	Website
Rotondi et al [45]	United States	Persons with schizophrenia or schizoaffective disorders (n=31) and their informal caregivers	24	63/F	Weak	Website
Ruskin et al [52]	United States	Caregivers of n=22 patients with schizophrenia or schizoaffective disorder	16	Not reported	Weak	eMonitor
Sin et al [53]	United Kingdom	Siblings of 18 people with psychosis	19	84/F	Weak	Website

^a^QATO: Quality Assessment Tool for Quantitative Studies.

^b^F: female.

**Table 2:** Study aims, intervention components and findings of reviewed studies.

Reference	Study aim	Key intervention components	Main findings
Chan et al [47]	Usability of an internet-based Psychosis Education Program designed to provide up-to-date and interactive online information about psychosis and local resources	Information about psychosis, caregiver coping, and support Information on local resources Downloadable video and written information Interactive discussion with caregiver peers and professionals	(1) On average, participants used website 2-5 times; (2) 85.2% reported improved knowledge about psychosis; (3) 74.7% felt supported by the site; (4) >80% would recommend the website to others; (5) 80% felt website was easy to use; (6) 81.5% felt website had sufficient information
Glynn et al [48]	Feasibility and quasi-experimental 12-month trial of the online multifamily group program for relatives of persons with schizophrenia	Discussion board Resources links Psychoeducational videos and information Interactive live chat between participants and professionals on Sunday evenings to focus on problem solving, illness management concerns Optional groups focused on Medication Social support	(1) 79% of caregivers completed the intervention; (2) 52.6% attended core Sunday evening sessions. 84.6% used the discussion board; (3) 30% engaged with optional groups; (4) 92% expressed satisfaction with the intervention; (5)14% reported having initial difficulty with the website; trend significance for patients from experimental group to be hospitalized less during the year of the intervention (24% vs 50%, *X*^2^_1_=2.9, *P*<.09)
Haley et al [49]	To evaluate the effectiveness of caregiver psychoeducation course delivered via telepsychiatry	6x2-h educational sessions Sessions delivered by interactive videoconferencing equipment	Significant increases at postintervention in caregiver knowledge about psychosis
Ozkan et al [50]	Randomized controlled trial to assess the impact of psychoeducation in a hospital clinic and telepsychiatric follow-up after inpatient discharge	Initial 8 sessions of face-to-face psychoeducation followed by 6 months of regular 15-min phone calls from clinician on set days to facilitate the expression of emotion	Significant postintervention, reduction in the experimental group in levels of caregiver burden, expressed emotion, and depression (*P*<.001)
Perron [51]	To examine an online self-help group for caregivers of people with mental health problems specifically the patterns and functions of their communications	An open asynchronous group comprising email and bulletin board	Participants posted an average of 12.6 messages (range, 1-92); male participants posted an average of 1.8 messages versus females who posted an average of 15.5; disclosure (eg, about their participant’s lives, their emotions, their relative’s condition) was the most common type of message function
Rotondi et al [46]	Randomized controlled trial evaluating the feasibility of random allocation to Schizophrenia Online Access to Resources website intervention delivering online multifamily therapy for persons with schizophrenia and caregivers; 3-month outcomes	Three professionally led facilitated therapy forums that were for patients only, caregivers only, and patients and caregivers together Therapy forums focused on problem solving, stress management, and peer support Ask the “Expert” questions Educational resources library, including information on local events and relevant news	(1) Therapy groups were the most used component of the website by caregivers; (2) no significant differences in outcomes between caregivers in experimental and treatment as usual groups but patients reported significantly less perceived stress; (3) 27.3% of caregivers reported loneliness when using the website; (4) caregivers suggested areas for the improvement included the greater provision of medication information and research on treatments, and the inclusion of website areas for caregivers to communicate about nonpsychosis-related issues (eg, cooking recipes)
Rotondi et al [45]	Examine use and benefits following random allocation to the Schizophrenia online access to resources website, delivering online multifamily therapy; 12-month outcomes	Three professionally led facilitated therapy forums that were for patients only, caregivers only, and patients and caregivers together Therapy forums focused on problem solving, stress management, and peer support. Ask the “Expert” questions Educational resources library, including information on local events and relevant news	(1) 92% were engaged in the treatment program; (2) caregivers spent an average of 14 h on the site (range, 30-4021 min) and were in active therapy for an average of 9 months (range, 1-19); (3) significant improvement in caregivers’ knowledge of psychosis, specifically, beliefs about prognosis
Ruskin et al [52]	Feasibility of using a home-based computerized device (Med-eMonitor) to enhance the monitoring of patient medication compliance and symptoms and to provide psychoeducation over a 2-month period	Med-eMonitor records the date and time of when patient medication containers are opened and records when medications are missed Prompts medication compliance through emitting an audible tone Liquid crystal display screen that provides factual information on psychosis and poses questions to patients and caregivers about patient clinical status	(1) Caregivers reported significant improvement in knowledge about psychosis (*t*=2.39, *P*=.05) but no improvements noted in the patient sample; (2) caregivers believed the monitor impacted positively on patients taking their medication more regularly and helped the caregiver remember to give medication; (3) caregivers reported satisfaction with the monitor though most would opt not to use the monitor after the end of the study
Sin et al [53]	Evaluate user satisfaction and usability over a 4-week period of an online psychoeducation intervention for siblings of people with psychosis “E siblings”	Downloadable factual information about psychosis Ask the Experts Direct access to advice from 12 professionals Interactive modules on self-care and coping Frequently Answered Questions Links to resources Discussion forum and blog	(1)17 participants completed the full evaluation; (2) participants each spent approximately 2 h (SD 72 min) using the site. Average site visits were 25 mins (SD 12); (3) all participants rated intervention highly, and approximately 95% rated content as very relevant to them; (4) 88.2% rated the intervention as being helpful; (5) 70.5% would recommend the site to others

##### Length of Intervention

The duration of the intervention under review was not always clear. No information was offered in Chan et al’s study [47], while others described interventions that lasted from 1 month [53] to 12 months [45,48]. Comparisons among studies were difficult because the amount of exposure to the given intervention was often evaluated and reported in different ways. While some papers provided data on the total number of minutes a participant was engaged in the intervention [45,47,53], others described the percentage of the sample who engaged with different components of the intervention [48].

#### Intervention Types

Variability was present in the digital interventions described and their intended outcomes. While 5 studies could be best described as Web-based [45-48,53], 2 studies used a telepsychiatry approach comprising a phone [50] or videoconferencing [49]. The remaining two studies used an eMonitor [52] or combined email and bulletin board [51].

##### Web-Based

In two of the Web-based studies [47,53], the intervention focused on the provision of information and tailored resources to support caregivers with more informed understanding of psychosis. Chan et al [47] reported data on perceptions of the usefulness and ease of use from 81 participants who accessed an internet-based psychoeducation program for first-episode psychosis caregivers in Hong Kong. The program sought to provide relevant and updated information using downloadable text-based papers, talking head videos (ie, where someone talks directly to the camera) from experts on different aspects of psychosis (eg, cause, treatments, and relapse), caregiving (eg, coping strategies and self-care), and local resources (eg, residential care services and financial services). Information was available in large font size and delivered in English and Cantonese to appeal to and address the needs of a broad group of users. Moreover, the Web platform provided a Web-based and interactive forum, which was moderated and designed to support discussion among peers and between peers and clinicians. Caregivers could post questions and receive responses from clinicians and other caregivers.

Sin et al [53] assessed the usability and feasibility for 16 participants accessing a Web-based platform exclusively dedicated to addressing the information and support needs of siblings of people with psychosis. The platform included 4 main components that focused on the information provision about the illness, coping and well-being, peer discussion forums and blogs, and an “ask the (clinical) expert” feature. The latter allowed participants to post questions to dedicated health care professionals (eg, general practitioner, mental health nurse, and psychiatrist) and receive tailored responses.

The remaining 3 papers, which described 2 studies [45,46,48], used a Web-based platform to deliver family intervention therapies that hitherto were typically implemented through face-to-face meetings held in clinics. Glynn et al [48] completed a proof-of-concept open trial, using a quasi-experimental design, to evaluate the feasibility of a 12-month online multifamily group intervention designed to provide education and support.

In their study, 26 caregivers of community-dwelling patients were involved in the intervention and data were compared with a historical treatment as usual sample (n=16). Caregivers had to have access to computers at their home to be eligible to participate; the Web program also comprised a discussion board, links to relevant resources, and educational videos. Participants were organized into small groups of 5-6 caregiver participants. During 1 year (comprising 6 months on a weekly basis, and biweekly for further 6 months), participants could access an hour-long educational talk on problem solving, and goal setting sessions with a psychologist and a research staff member. These sessions were held on Sunday evenings. Furthermore, caregivers could access additional groups on medication and support. The authors noted their small sample size despite “intensive” recruitment strategies implemented. That said, participants reported high levels of satisfaction (>90%) with the intervention, with 84.6% engaging with the discussion board, 52.6% with the Sunday talk sessions, and one-third attending the additional groups. No impact of the intervention was observed on the levels of caregivers’ reported distress (or patient functioning). There was a trend of significance that the intervention was linked to fewer patient admissions during the intervention year.

Rotondi et al [45,46] completed a randomized feasibility trial of multifamily psychoeducational interventions also using a Web platform. Unlike Glynn et al [48], in this study, participants were issued computers in their home, if required. In addition, 21 caregivers of inpatients and community-dwelling patients, with a history of at least 1 hospital admission in the preceding 2 years were randomly assigned to the Web intervention or treatment as usual. In the intervention arm, caregivers were issued with a unique log-in name (which was not allowed to be their real name) and password access to a caregiver-only website therapy group and a joint group designed for caregivers and patients together. The therapy group focused on problem solving and offered a bulletin board for communication among group members. The groups were led by mental health professionals and guided by therapy manuals [54,55]. As part of additional intervention modules, caregivers were given opportunities to anonymously pose questions to experts, to receive responses to their questions, and to view questions asked by other caregivers and responses they received. Moreover, they had access to relevant reading material and local relevant mental health news. Before commencing the Web-based intervention, all participants (caregivers and patients) were required to attend a joint 4-hour psychoeducation workshop. Furthermore, outcome data were collected at 3, 6, and 12 months. The most used intervention components within the platform were the two therapy groups (ie, caregiver-only group and caregiver and patient group). The authors failed to identify any difference in outcomes between caregivers in the experimental and treatment arms; however, the patient group reported markedly less stress. Nearly one-third of caregivers reported feeling lonely in their use of the website.

##### Telepsychiatry

In terms of the two telepsychiatry studies, both focused on the provision of psychoeducation to improve caregivers’ knowledge and understanding of the illness and to promote more effective coping strategies. Haley et al [49] delivered a psychoeducation course to 56 caregivers in Dublin and Donegal regions of Ireland using an interactive videoconferencing system that included a Tandberg Director, camera, plasma monitors, and three Integrated Services Digital Network lines. Participants recorded a marked increment in their knowledge postintervention. Ozkan et al [50] used a randomized controlled design to evaluate the impact of providing short (ie, 15 minutes) telephone calls over a 6-month period to caregivers. Caregivers received these calls following their relative’s discharge from hospital and following their (ie, caregiver) own participation in an 8-session, face-to-face psychoeducation intervention during the admission. The calls were designed to focus on caregivers’ emotions and their experience of burden. The intervention group recorded markedly lower rates of caregiver burden, depression, and emotional expression.

##### Email or Bulletin Board

Ruskin et al [52] assessed the feasibility of using a computer-based device “Med-eMonitor,” which could be preprogrammed to ask questions and display illness-related factual information (eg, schizophrenia incidence rates), to assist patients in improving their daily medication adherence and provide caregivers with psychoeducation. The study duration was 2 months, and 16 caregivers used the monitor to get responses to questions about how they understood psychosis (eg, symptoms). Marked improvements were noted in caregivers’ understanding about the illness and their ability to remind their relatives to take their medications. However, they preferred not to use the monitor once the study period ended.

Perron [51] presented data from 33 participants who engaged in an online self-help group for mental health caregivers over an 18-month period. It was an open group (ie, free and open to the general public to engage with), with no moderator and organized around email exchanges and bulletin boards. Perron [51] analyzed the content of 417 messages posted within 18 months. Participants posted an average of 12.6 messages. Male participants posted fewer messages than with female participants (average, 1.8 vs 15.5). The most common category for a posted message was “disclosure.” These were messages where participants tended to provide updates on their lives, their experiences with their relative (eg, treatment-related issues and symptoms), and their own emotions. Other key functions identified from posted messages focused on the provision of information, support, and empathy to caregiver peers. Only one message’s content was rated as being negative, which did not receive any replies or comments from other caregivers.

### Methodological Quality

Overall, 7 papers were scored weakly on the overall QATO rating; these had weakness in the study design, approaches to data collection, management of confounding variables, and assessor independence. In addition, 2 studies [48,50] obtained an overall QATO rating of moderate and had a stronger study design (eg, randomized controlled trial or quasi-experimental) and more robust participant selection and data collection approaches (Table 1).

## Discussion

### Principal Findings

The literature highlights an increasing interest in and documentation of digital technologies in health care and their novel applications with psychosis disorders. Improved patient outcomes in psychosis rely heavily on the input from informal caregivers. This systematic review explored the application and outcomes of digital technologies in informal caregivers of people with psychosis.

The review yielded 9 papers reporting data from 8 independent studies. Overall, two-thirds of the included studies focused solely on the recruitment and outcomes of caregivers only and one-third recruited caregiver and patient dyads. The reviewed studies were diverse, with origins in Europe, Central and North America, Asia, and the Middle East, in reflecting the global developments in digital technologies. However, most studies were from the United States, which might reflect different health care priorities that predominate in that region and broader developments in digital innovations. As an estimated 89% of the US population accesses the internet [56], evaluating the contribution of digital interventions to improving outcomes in caregiver groups might seem a sensible development.

There was also diversity in the digital applications under review. The interventions included telepsychiatry and email. Most studies were, however, best described as being Web-based and included those with a dedicated platform with functionality to facilitate communication between caregiver groups and caregivers and professionals. Remarkably, no study identified detailing the use of mobile apps and virtual or augmented realities, which contrasts markedly with developments the literature has observed in psychosis patient populations [57,58]. There is no evidence suggesting that caregivers would be any less likely than peers and other groups to take advantage of mobile phone apps or relevant health-focused augmented realities. A general commitment to exploring and investing in opportunities to expand the range of digital approaches on offer or applicable to caregivers should be prioritized to minimize gaps in service provision and the potential for a digital divide between caregivers and others.

The review findings indicate that we are far from being in a position to offer definitive data about the use and impact of digital innovations in caregivers. The majority of studies under review were described from the outset as being studies of the feasibility and usability. Though studies provided useful data, in the absence of powered experimental designs, definitive conclusions about outcomes and effects are premature.

In the majority of studies, the reason underlying the development and use of the digital tool was the provision of relevant information about psychosis (psychoeducation), delivered as part of a structured educational course or through different independent and related modules. A key component in treatment recommendations for caregivers of people with psychosis [23] is information on how to best understand the illness and facilitate the use of adaptive and effective coping strategies. Psychoeducation is a need commonly reported by caregivers [59], but an area that is often unmet by service providers [60,61]. Over the course of the illness, caregivers will often be expected to make sense of illness-related information that can at times be complex, confusing, and vague. For many, this information will be given during periods when they are also experiencing high levels of stress and therefore perhaps more likely to benefit from varied methods of sharing the relevant information. Though preliminary, the early indications from the findings suggest that caregivers’ understanding about psychosis might benefit from the use of digital technologies and that the approach might be acceptable [45,47,49]. Further exploration of the benefits of using different approaches to support caregivers in facilitating their knowledge and understanding of their relative’s mental health condition, which also extends beyond traditional face-to-face meetings [62], would represent important research developments and progress in the field.

We do, however, remain aware that at least one-third of caregivers in one website study reported feelings of loneliness in using the intervention [46], and in another study [52], caregivers would opt not to continue using the digital equipment when the study ended. However, at this stage, it remains unclear as to what extent these types of findings generalize and form a distinct pattern. Hence, further evaluations utilizing quantitative and qualitative investigations are indicated. Though the interest in and appetite for digital technologies in mental health sectors remains on a steady upward trend, these findings also underscore the importance of seeking to identify caregiver subgroups for whom digital approaches might not always suit and to address their specific presenting needs.

This study suggests a lack of uniformity in terms of the key areas to measure as outcomes. It is also noteworthy that the website and email or bulletin intervention studies included components that promoted and allowed for peer interactions and support. However, measures of social support and social networks were absent. The current evidence attests that psychosis caregivers are up to 10 times more socially isolated than the general population and typically fair worse in terms of support levels when compared with caregivers of adults with similar challenging conditions [63]. It is important to extend the digital technology outcome literature beyond the rates of take-up and satisfaction. The results indicate a need for further work to be undertaken to identify target outcome areas for measurement in the use of digital technologies with caregivers and the preferred methods of assessment, which in turn could lead to more meaningful evaluation of studies and comparison of findings.

Notably, however, there were 2 studies that evaluated a telepsychiatry [50] and website-based intervention [48] that obtained overall moderate ratings; this reflected their superiority in the study design (eg, randomized controlled trial or quasi-experimental) and participant selection and data collection approaches.

### Limitations

The focus of the review on caregivers of people with psychosis is a strength of this study and is in line with other reviews focusing on technology in different caregiver populations such as older adults [64] and severe mental illnesses [65]. The review, however, does have some limitations. First, the studies were mainly investigations of feasibility, usability, and acceptability and were therefore not designed or powered to offer definitive conclusions about the efficacy and direction of findings, which largely reflects the poor methodological quality ratings. The majority of papers (n=7) obtained weak QATO ratings that were representative of a broad range of issues reflecting inherent difficulties in the study design, approaches to data collection, management of confounding variables, and assessor independence. However, given that some might argue about the relative infancy of the literature and the predominance of study designs (eg, usability and feasibility), the ratings might simply reflect the stage of the literature with stronger and more methodologically sound studies to follow.

Second, a modest number of studies were under review and the overall participant size was small. The smaller studies reported samples of n=16. Notably, the recruitment pathways for some studies were dependent on both patient and caregiver consenting to participate [48], while others could directly recruit and consent caregivers [53]. The different recruitment approaches across studies are likely to have implications for the sample and their presenting needs. For example, studies that required patient and caregiver consent might be more likely to recruit groups who were better functioning or had better quality caregiving relationships, which arguably is likely to impact their engagement. The majority of studies were from the United States, a high-income nation with large sections of the adult population accessing the internet. The review was limited to English language publications. Consequently, the interpretation of findings and their generalizability to other settings and communities are limited. Furthermore, while we may have sought to be overinclusive in our search approach, it remains possible that we might have missed potentially relevant studies, given the language restrictions and parallel exclusions of case studies and qualitative investigations. Not dissimilar to other systematic reviews, it is possible that our review will be subject to publication bias because nonsignificant findings are less likely to be published. Therefore, the reviewed studies could overrepresent the positive effects of digital interventions with caregivers.

### Conclusions

Notwithstanding the continued value of direct service input and face-to-face contact, the potential contribution of digital interventions to impacting outcomes for psychosis caregivers and addressing their specific needs for information, support, and well-being deserves greater clinical and research interest. Evidently, given the number and range of studies reviewed, widely established digital developments witnessed in patient mental health care have yet to be replicated in caregiver populations. However, this study offers preliminary support that these types of interventions (eg, Web-based) can be feasible and acceptable to caregivers. Much further development in the range of technologies on offer and robust evaluation of their outcomes, including cost-effectiveness [66], is required. Furthermore, the study indicates the inclusion of caregiver-reported outcomes and their qualitative reports of satisfaction.

## Abbreviations

QATO: Quality Assessment Tool for Quantitative Studies
